# Supplementary material for: Identifying Calmodulin and Calmodulin-like Protein Members in Canavalia rosea and Exploring Their Potential Roles in Abiotic Stress Tolerance
Source: Int J Mol Sci. 2024 Oct 31;25(21):11725. doi: 10.3390/ijms252111725 (PMC11545983; doi:10.3390/ijms252111725)
Supplement: Supplementary file 1 [file ijms-25-11725-s001.zip › Appendix A. Supplementary data-ijms-3259335-final version--20241028.pdf]

Supplementary Material Information

Identifying calmodulin and calmodulin-like protein members in *Canavalia rosea* and exploring their potential roles in abiotic stress tolerance

Qianqian Ding [1, 2, †], Zengwang Huang [1, 2, †], Zhengfeng Wang [1, 3, 4], Shuguang Jian [1, 3] and Mei Zhang [1, 3, *]

Citation: To be added by editorial staff during production.

Academic Editor: Firstname Lastname

Received: date

Revised: date

Accepted: date

Published: date

Copyright: © 2024 by the authors. Submitted for possible open access publication under the terms and conditions of the Creative Commons Attribution (CC BY) license

(https://creativecommons.org/licenses/by/4.0/).

[1] Guangdong Provincial Key Laboratory of Applied Botany&South China Agricultural Plant Molecular Analysis and Genetic Improvement, South China Botanical Garden, Chinese Academy of Sciences, Guangzhou 510650, China;

[2] University of the Chinese Academy of Sciences, Beijing 100039, China;

[3] CAS Engineering Laboratory for Vegetation Ecosystem Restoration on Islands and Coastal Zones, South China Botanical Garden, Chinese Academy of Sciences, Guangzhou 510650, China;

[4] Southern Marine Science and Engineering Guangdong Laboratory (Guangzhou), Guangzhou 511458, China;

[#] These two authors contributed equally.

* Correspondence and requests for materials should be addressed to Mei Zhang (zhangmei@scbg.ac.cn).

Figure S1. The 3D models of 51 CrCaMs/CrCMLs constructed using Phyre2 (http://www.sbg.bio.ic.ac.uk/~phyre2/html/page.cgi?id=index).

Table S1. Primer lists used in this study and the sequences information of *CrCaMs*/*CrCMLs*' genomic DNA, CDS, and promoter region DNA.

Table S2. *Cis*-acting elements (CEs) analysis of *CrCaMs*' and *CrCMLs*' promoters.

Table S3. FPKM values of *CrCaMs*/*CrCMLs* for RNA-Seq assay of *C. rosea* tissues in this study.

Fig. S1

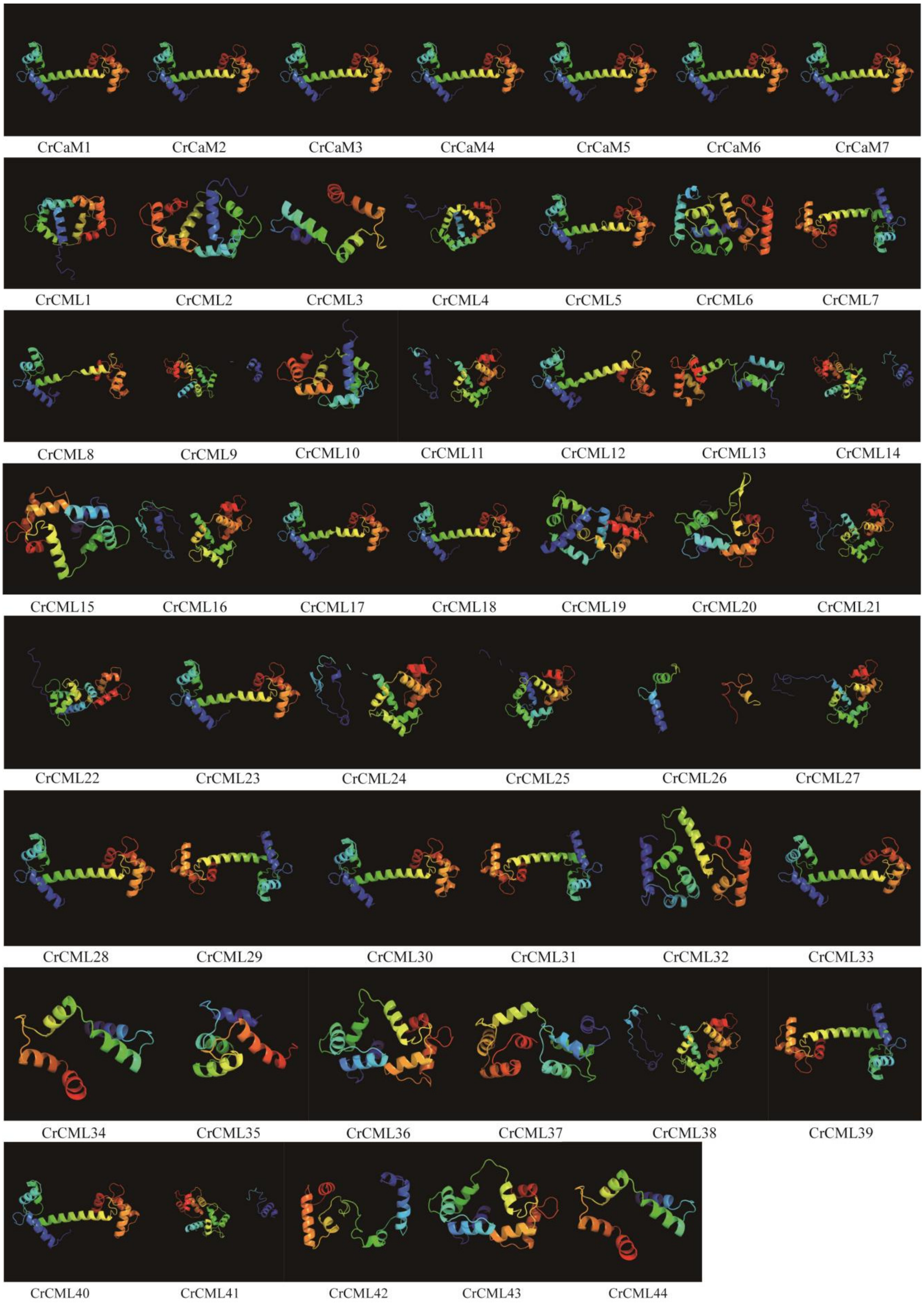
CrCaM1 CrCaM2 CrCaM3 CrCaM4 CrCaM5 CrCaM6 CrCaM7
CrCML1 CrCML2 CrCML3 CrCML4 CrCML5 CrCML6 CrCML7
CrCML8 CrCML9 CrCML10 CrCML11 CrCML12 CrCML13 CrCML14
CrCML15 CrCML16 CrCML17 CrCML18 CrCML19 CrCML20 CrCML21
CrCML22 CrCML23 CrCML24 CrCML25 CrCML26 CrCML27
CrCML28 CrCML29 CrCML30 CrCML31 CrCML32 CrCML33
CrCML34 CrCML35 CrCML36 CrCML37 CrCML38 CrCML39
CrCML40 CrCML41 CrCML42 CrCML43 CrCML44

Tab. S1

Table S1.xlsx

Tab. S2

Table S2.xlsx

Tab. S3

Table S3.xlsx
