# Supplementary figures and images for: Identifying Calmodulin and Calmodulin-like Protein Members in Canavalia rosea and Exploring Their Potential Roles in Abiotic Stress Tolerance
Source: Int J Mol Sci. 2024 Oct 31;25(21):11725. doi: 10.3390/ijms252111725 (PMC11545983; doi:10.3390/ijms252111725)

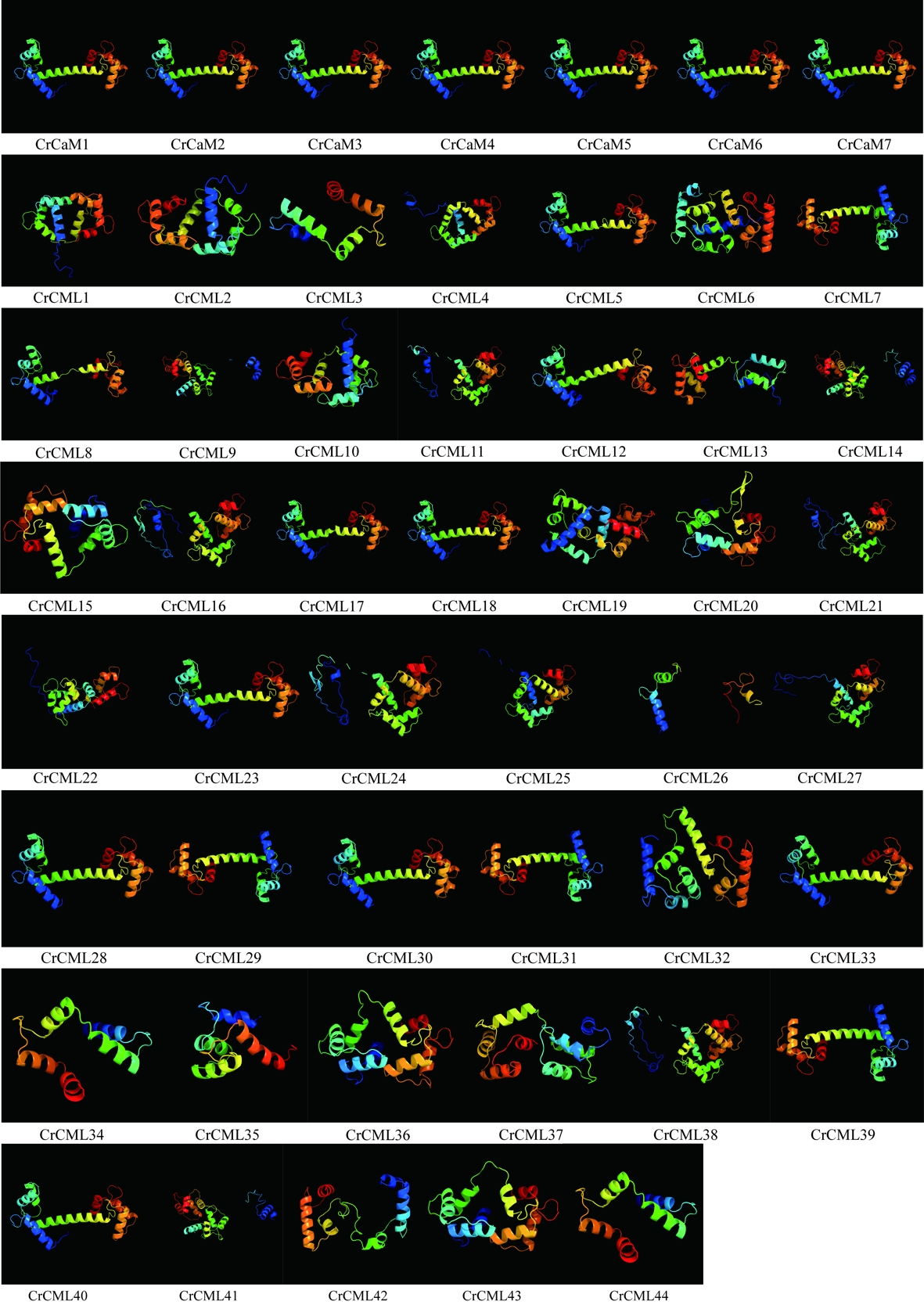

Supplement: Supplementary file 1 [file ijms-25-11725-s001.zip › Figure S1-ijms-3259335-final version--20241028.jpg]
